# A Reduction in Antenatal Steroid Dose Was Associated with Reduced Cardiac Dysfunction in a Sheep Model of Pregnancy

**DOI:** 10.1007/s43032-023-01264-2

**Published:** 2023-06-01

**Authors:** Yusaku Kumagai, Matthew W. Kemp, Haruo Usuda, Tsukasa Takahashi, Yuki Takahashi, Hirotaka Hamada, Augusto F. Schmidt, Takushi Hanita, Shimpei Watanabe, Shinichi Sato, Hideyuki Ikeda, Erin L. Fee, Lucy Furfaro, John P. Newnham, Alan H. Jobe, Nobuo Yaegashi, Masatoshi Saito

**Affiliations:** 1 Centre for Perinatal and Neonatal Medicine, Tohoku University Hospital, Sendai, Miyagi Japan; 2 Division of Obstetrics and Gynecology, The University of Western Australia, Perth, WA Australia; 3 College of Veterinary and Life Sciences, Murdoch University, Perth, WA Australia; 4 Department of Obstetrics and Gynaecology, Yong Loo Lin School of Medicine, National University of Singapore, Singapore, Singapore; 5 University of Miami, Miami, FL USA; 6 Cincinnati Children’s Hospital Medical Centre, Cincinnati, OH USA

**Keywords:** Antenatal corticosteroid, Betamethasone phosphate, Fetal programming, Cardiac function, Hypertrophy of cardiomyocytes, Developmental origins of health and disease (DOHaD)

## Abstract

Despite widespread use, dosing regimens for antenatal corticosteroid (ACS) therapy are poorly unoptimized. ACS therapy exerts a programming effect on fetal development, which may be associated with an increased risk of cardiovascular disease. Having demonstrated that low-dose steroid therapy is an efficacious means of maturing the preterm lung, we hypothesized that a low-dose steroid exposure would exert fewer adverse functional and transcriptional changes on the fetal heart. We tested this hypothesis using low-dose steroid therapy (10 mg delivered to the ewe over 36 h via constant infusion) and compared cardiac effects with those of a higher dose treatment (30 mg delivered to the ewe over 24 h by intramuscular injection; simulating currently employed clinical ACS regimens). Fetal cardiac function was assessed by ultrasound on the day of ACS treatment initiation. Transcriptomic analyses were performed on fetal myocardial tissue. Relative to saline control, fetuses in the higher-dose clinical treatment group had significantly lower ratios between early diastolic ventricular filling and ventricular filling during atrial systole, and showed the differential expression of myocardial hypertrophy-associated transcripts including βMHC, GADD45γ, and PPARγ. The long-term implications of these changes remain unstudied. Irrespective, optimizing ACS dosing regimens to maximize respiratory benefit while minimizing adverse effects on key organ systems, such as the heart, offers a means of improving the acute and long-term outcomes associated with this important obstetric therapy.

**Supplementary Information:**

The online version contains supplementary material available at 10.1007/s43032-023-01264-2.

## Background

Preterm birth, defined by the World Health Organization (WHO) as delivery before 37 weeks’ completed gestation, is a leading cause of neonatal death. Antenatal corticosteroid (ACS) therapy is a well-established intervention for women at risk of preterm delivery between 24 and 34 weeks of gestational age, and significantly reduces the risk of perinatal death and respiratory distress [1]. The accelerated fetal lung maturation elicited by ACS exposure is crucial for reducing neonatal morbidity and mortality [2]. In addition, the vasopressor activity of ACS has the potential to stabilize fetal hemodynamics, acting synergistically with the primary effect of improved respiratory function [3].

However, numerous studies have identified a potential risk of longer-term adverse effects associated with ACS, such as growth restriction [4, 5], cognitive deficits [6, 7], mental and behavioral disorders [8, 9], elevated stress responses [10], altered insulin responsiveness in the adult [11], and possible epigenetic/transgenerational effects [12]. Adverse intrauterine exposures, such as prenatal glucocorticoid administration, can alter the structure, homeostatic systems, and functions of the body [13, 14]. This phenomenon, referred to as fetal programming, has been explored in work pertaining to the developmental origins of health and diseases (DOHaD) theory [15].

Despite ACS being used clinically for nearly 50 years, dosing to optimize benefits and minimize harms remains largely unexplored in the clinic [1, 2, 16]. Using a sheep model, we have previously shown it is possible to induce fetal lung maturation using substantially lower ACS exposures than those employed in current clinical dosing regimens.

Previous studies from our group demonstrated that a fetal plasma exposure of only 2 ng/mL betamethasone for 26 h was sufficient enough to induce lung maturation as early as day 2 of treatment [17]. In these studies, high peak maternal and fetal plasma levels of betamethasone were not necessary for the maturation effects of ACS. Similar findings were demonstrated in extended-duration, 36-h betamethasone phosphate (Beta-P) (Betnesol, Focus Pharmaceutical, London, UK) exposures [18].

With regard to the cardiovascular system, and the myocardium in particular, elevated levels of glucocorticoids have been linked to a variety of negative cardiac outcomes. For example, excessive in utero exposure to glucocorticoids can have a programming effect and lead to an increased risk of cardiovascular disease later in the adulthood [19–22]. Moreover, ACS use is associated with decreased heart rate variability at 14 years of age in Caucasian female subjects relative to a well-matched comparison group of children with birth weights of about 1 kg [23], and multiple studies have reported that glucocorticoids induce cardiac hypertrophy, which is a major cause of congestive heart failure [24, 25].

In the present study, we elected to focus on the acute in utero effects of ACS on the fetal heart. We hypothesized that a low-dose steroid exposure would exert fewer adverse functional and transcriptional changes on the fetal heart. When assessed against earlier work, this study will allow us to assess the potential to use lower fetal steroid exposures to minimize off-target or adverse effects on key fetal organs (such as the heart) while still achieving robust lung maturation necessary to reduce the risk of respiratory distress syndrome and neonatal death in preterm infants.

## Methods

### Overview of Experiments

The pulmonary outcomes for animals in this study have previously been published [18]. Pharmacokinetic data obtained previously was revised and is shown in Supplementary Fig. 1 [17]. In the present study, a randomly selected subgroup of six animals from the three treatment groups were chosen for cardiac function and transcriptome analysis. Two competing constraints informed our selection of group sizes—uncertainty regarding the size of potential cardiac effects detectable by ultrasound, and existing data showing glucocorticoid effects are strongly time-bound, hence the need for tightly standardized experiments. Accordingly, we elected to use a smaller number of tightly controlled animals (in terms of treatment to ultrasound scan interval and time of day assessed) to minimize experimental noise. A post hoc power analysis of E/A ratio differences showed that the power (1-β err prob) of MV-E/A, TV-E/A is 0.55, 0.77 respectively. Fetal cardiac function in the acute treatment phase was assessed by ultrasound 8 h after ACS treatment initiation. This time point was selected to correspond with the anticipated maximum concentration for the clinical high-dose treatment, standardizing measurement time. Such an approach is justified given data showing an association between glucocorticoid effects (including on peripheral resistance and blood pressure [28]) and magnitude of drug exposure, and the fore-mentioned impacts of treatment to assessment interval. Lambs were surgically delivered 7 days after treatment commenced for subacute evaluation. We examined the effects of ACS on gene expression changes in fetal myocardium in these animals.

### Animal Work

Animal experiments were performed in Perth, Western Australia, following review and approval by the Animal Ethics Committee, University of Western Australia (approval RA/3/100/1378), and the Animal Ethics Committee, Murdoch University (approval R3056/18). All pregnant sheep were sourced from a single supplier, and experiments were performed during the normal breeding season. Ewes carrying a single fetus received an intramuscular injection of 150-mg medroxyprogesterone acetate (Depo-Ralovera®; Pfizer, West Ryde, NSW, Australia) to reduce the risk of preterm labor. Five days later, sheep were randomized to either:

(i) A low-dose maternal intravenous infusion of betamethasone phosphate to target fetal plasma betamethasone concentrations of 2 ng/mL for 36 h;
(ii) A clinical course of two maternal intramuscular injections of 0.25 mg/kg Celestone Chronodose® (1:1 mixture of betamethasone phosphate and betamethasone acetate) spaced at 24 h; or
(iii) Maternal intramuscular saline as control. Six animals from each group were selected at random for cardiac ultrasound and transcriptomic analysis.

### Treatment Groups

**Fig. 1:**
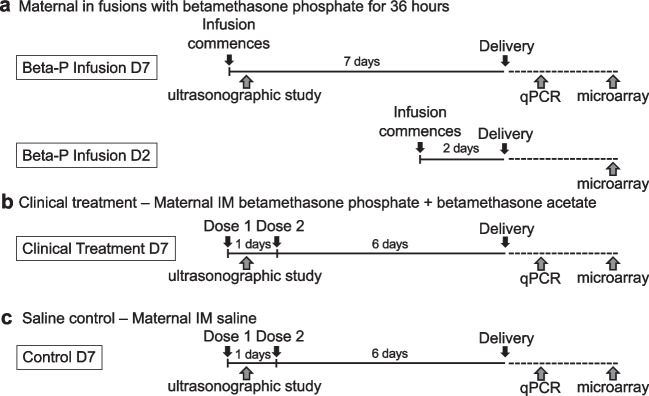
Schematic representation of study groups. **a** Two groups of pregnant ewes had maternal catheters placed and received an intravenous loading dose followed by a 36-h infusion of low-dose betamethasone phosphate, with delivery of preterm lambs at 2 or 7 days after treatment initiation. **b** One group of pregnant ewes were randomized to receive 2 doses of intramuscular dose of 0.25 mg/kg maternal weight betamethasone phosphate and betamethasone acetate separated by 24 h, with delivery of preterm lambs at 7 days after treatment initiation. **c** Saline-treated animals served as controls

Treatment protocols are summarized in Fig. 1.

Three groups of animals underwent combined ultrasound and transcriptome analyses. For the betamethasone phosphate infusion group animals, ewes had external jugular catheters placed under general anesthetic as published previously [26]. Two days later, animals received an intravenous loading dose followed by constant infusion of betamethasone phosphate to achieve maintain 2 ng/mL fetal plasma betamethasone concentrations (0.028 mg/kg intravenous bolus + 0.144 mg/kg/36 h intravenous infusion) as previously described (Beta-P Infusion D7, *n* = 6) [18]. For the clinical treatment group animals, ewes received two maternal intramuscular injections of 0.25 mg/kg betamethasone phosphate + betamethasone acetate (Celestone Chronodose, Merck, Sharp and Dohme, Macquarie Park, NSW, Australia) spaced by 24 h (Clinical Treatment D7, *n* = 6). For the control group animals, ewes received maternal intramuscular saline solution at 114 days gestational age (Control D7, *n* = 6). Animals in these three groups were delivered 7 days after treatment initiation.

To allow for microarray analysis of acute vs. subacute transcriptional (i.e., 2-day vs. 7-day) changes, we included cardiac tissues from a 2-day infusion treatment to delivery interval group. In this group, ewes underwent an identical surgery and dosing treatment as per the above 7-day betamethasone phosphate infusion group, but with animals delivered 2 days after treatment was initiated (Beta-P Infusion D2,* n* = 6). All animals in the study were delivered between 119 and 124 days gestational age.

### Cardiac Ultrasound

**Fig. 2:**
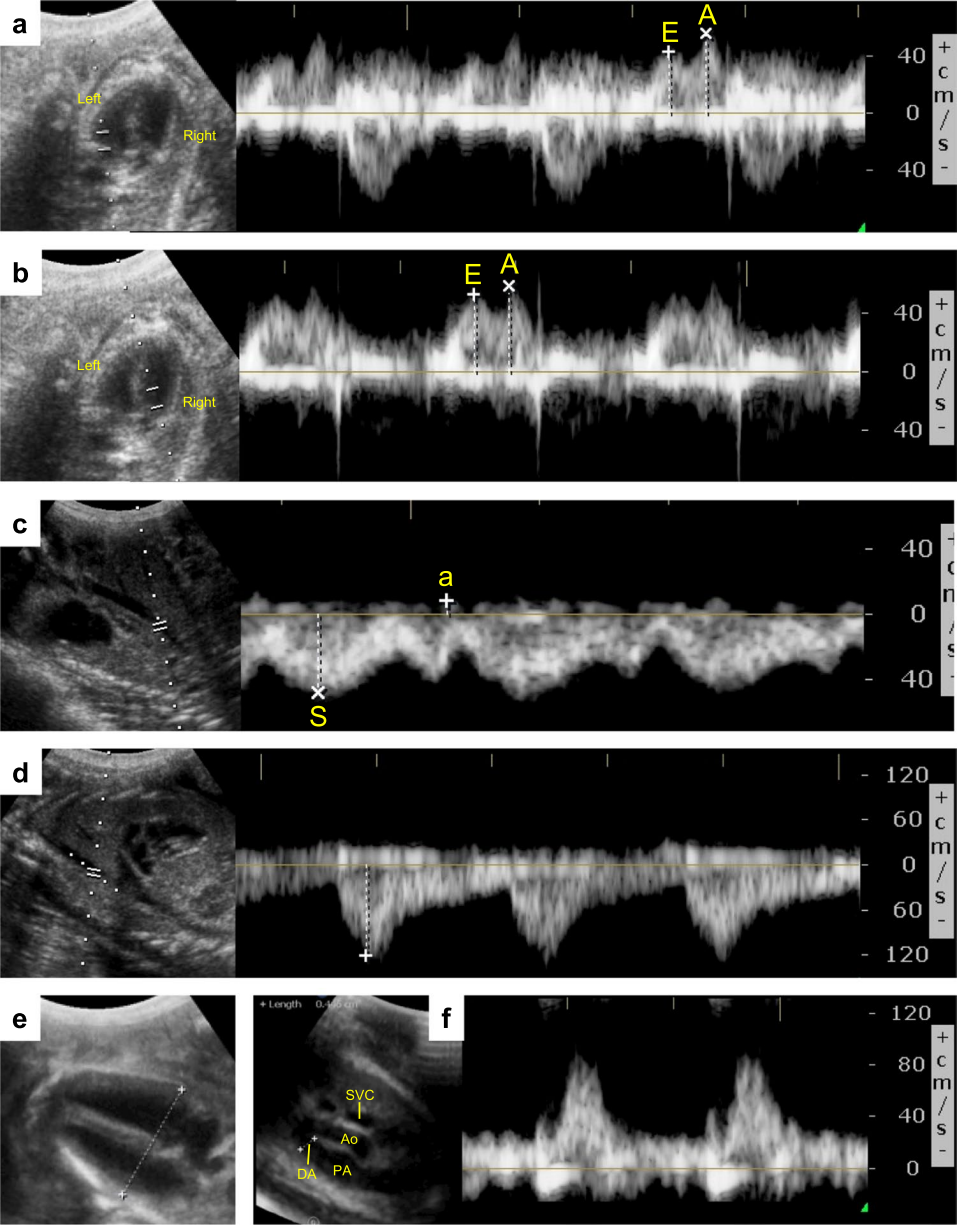
Representative images of fetal cardiac ultrasound. Cross-sectional view showing 4-chamber view (**a**, **b**, and **e**), sagittal view (**c** and **d**), and three-vessel trachea view (**f**). (A and B) Doppler waveforms across the mitral (**a**) and tricuspid (**b**) valves during diastole. In the biphasic doppler waveforms, E corresponds to early diastolic peak velocity and A corresponds to atrial contraction peak velocity. The E/A ratio is used for Doppler waveform quantification across the atrioventricular valves. **c** Doppler waveform of inferior vena cava. S represents maximum systolic velocity, and a represents the atrial reversal flow during atrial contraction. The a/S ratio was calculated to obtain the preload index. **d** Doppler waveform of descending aorta (dAo) at the level of the diaphragm. The maximum systolic blood velocity (Vmax) was measured. **e** Distance between the attachment point of the mitral valve on the epicardium to the attachment point of tricuspid valve on the epicardium was measured in the 4-chamber view as total cardiac dimension. **f** Doppler waveform of the ductus arteriosus. SVC, superior vena cava; Ao, aorta; PA, pulmonary artery

Ultrasound assessments were performed by a single operator 8 h after ACS treatments were initiated—approximating peak fetal exposure in both Infusion and Clinical Treatment Groups for reasons outlined above. Measurements were conducted with a Philips CX50 system, S5-1 phased-array probe (both Philips Healthcare, Best, The Netherlands) and associated obstetrics software. For animal measurements, ewes were held in dorsal recumbency and the fetal position from the ventral aspect was confirmed by an operator. The ultrasound beam was focused to obtain a basal 4-chamber view, 3-vessel trachea view, or sagittal view to check the six cardiac function parameters as shown below:

(i) The ratio of early diastolic peak velocity (E-wave) to atrial contraction peak velocity (A-wave) across the mitral valve (MV-E/A, Fig. 2a),
(ii) The ratio of E-wave to A-wave across the tricuspid valve (TV-E/A, Fig. 2b). The ratios between the E-and A-wave peak velocities are a generally accepted angle-independent index for quantification of the waveform,
(iii) Preload index of inferior vena cava (PLI, Fig. 2c);
(iv) The maximum peak velocity of descending aorta (dAo-Vmax, Fig. 2d);
(v) Transverse cardiac diameter (TCD, Fig. 2e); and
(vi) Doppler waveform of the ductus arteriosus (DA, Fig. 2f)

The angle of insonation between the ultrasound beam and the vessel/blood flow was within 60°.

### Necropsy

All animals were delivered by cesarean section under general anesthesia and were euthanized with intravenous pentobarbitone (160 mg/kg) after cord blood collection to measure blood pH, pO2, and pCO2. Left ventricular myocardial tissue samples were dissected and snap-frozen in liquid nitrogen before being stored at − 80℃ for further analysis.

### RNA Extraction

Total RNA was extracted using TRIzol (Invitrogen, Carlsbad, CA) according to the manufacturer’s protocol. RNA quality and integrity were verified using the Agilent 2100 Bioanalyzer (Agilent, Agilent Technologies, Santa Clara, CA).

### Quantitative Assessment of Gene Expression by Real-time PCR

Quantitative polymerase chain reaction (qPCR) cycling was performed with ovine-specific TAQMAN probe and primer sets (Applied Biosystems, Foster City, CA) with a Step One Real-Time PCR System in accordance with manufacturers’ instructions. Messenger RNA transcripts for the following eight cardiac targets were measured: MYH7_seq08593 (MYH7; XM_027977118.1; Oa04885566_m1); myosin-6 (LOC101120580)(MYH6; XM_027971620.1; Oa04758573_g1); growth arrest and DNA damage inducible gamma (GADD45G; XM_015093022.2; Oa04906954_g1); peroxisome proliferator activated receptor gamma (PPARG; NM_001100921.1; Oa04658387_m1); PPARG coactivator 1 alpha (PGC1-α; XM_004009738.4; Oa02631739_m1); and heat shock protein family A (Hsp70) member 5 (HSPA5; XM_004005637.3; Oa04856974_g1); natriuretic peptide B (NPPB; NM_001160026.1; Oa04931155_g1); nuclear receptor subfamily 3 group C member 1 (NR3C1; NM_001114186.4; Oa04657789_m1). Amplification data for each gene were normalized to ribosomal protein 18 s RNA as an internal reference.

### Microarray Analysis

Microarray analysis was performed on four groups: Control D7 group, Clinical Treatment D7 group, Beta-P Infusion D2 group, and Beta-P Infusion D7 group, with six biological replicates for each group. A total of 100 ng of total RNA was processed with a WTPlus Kit (Affymetrix) in accordance with manufacturer’s instructions. Fragmented, labelled, single-stranded DNA was hybridized with a GeneChip Hybridization, Wash and Stain Kit (Affymetrix) and submitted to an ovine-specific microarray analysis (Ovine Gene 1.1 ST 24 Array plate; Affymetrix, Santa Clara, CA), which includes 22,141 ovine genes to be analyzed. Array data were preprocessed using Transcriptome Analysis Console (TAC) 4.0.1 (Thermo Fisher Scientific Inc.) with the robust multiarray average algorithm, background correction, quartile normalization, and gene-level probe set summation. Differentially expressed genes (DEGs) (steroid treatments vs Control D7) were identified by 2-sample *t*-tests along with adjusted *p*-values for multiple testing using Benjamini–Hochberg false discovery rate (FDR) correction. FDR-corrected *p*-value lower than 0.1 was considered significant. Pathway analysis was performed using DAVID (Database for Annotation, Visualization and Interrogated Discovery) [27]. Significant terms were determined as FDR-corrected *p*-value < 0.05. Microarray analysis was performed by the Ramaciotti Centre for Genomics at the University of New South Wales.

### Statistical Analysis

Statistical analyses were performed with IBM SPSS for Windows, version 20.0 (IBM Corp, Armonk, NY). Mean differences between normally distributed data with equal variance were tested for significance with one-way analysis of variance (*p* = 0.05). The primary comparisons were for each treatment group against the Control D7 group, with Dunnett’s* t*-test.

## Results

### Biophysical Status of the Newborns

There was no difference between the experimental groups and Control D7 group in gestational age, birth weights, sex, or cord blood pH (Table 1).

**Table 1:** Control and treatment group delivery data

Treatments	*N*	Delivery GA (days)	Birthweight (kg)	Sex (male/female)	Cord pH
Beta-P Infusion D7	6	120.7 ± 0.3	2.6 ± 0.2	2/4	7.3 ± 0.0
Beta-P Infusion D2	6	122.5 ± 0.2	2.5 ± 0.4	2/4	7.3 ± 0.0
Clinical Treatment D7	6	122.5 ± 0.7	2.8 ± 0.3	2/4	7.3 ± 0.0
Control D7	6	121.2 ± 0.5	2.5 ± 0.3	4/2	7.3 ± 0.1

Values are expressed as mean ± one standard error

*Beta-P*, betamethasone phosphate

### Ultrasonographic Analysis of Cardiac Function

**Fig. 3:**
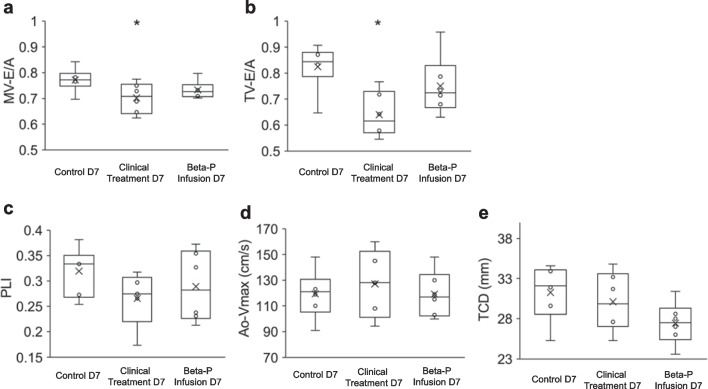
Differential fetal cardiac function by ultrasound. After 8 h, the first of two maternal intramuscular steroid doses administered to the Clinical Treatment D7 group (i.e., the first of two doses of 0.25 mg/kg betamethasone phosphate and acetate) decreased both of E/A ratio. After 8 of 36 h of betamethasone phosphate infusion in the 7-day betamethasone phosphate group, there were no statistically significant differences in fetal cardiac function detected. **a** Mitral valve E/A ratio. **b** Tricuspid valve E/A ratio. **c** Preload index of inferior vena cava. **d** The maximum systolic blood velocity (Vmax) of descending aorta. **e** Total cardiac dimension. *p < 0.05 vs. control

To assess the short-term effects of glucocorticoid exposures on fetal cardiac function, we performed ultrasonographic analysis on fetal cardiac system 8 h after the treatments were initiated. Pulse-Doppler imaging was used to detect the blood flow direction and wave form through the atrioventricular valves, inferior vena cava, descending aorta, and ductus arteriosus. The blood flow through the *ductus arteriosus* in all eighteen ultrasound-examined fetuses was right-to-left directional flow (from the pulmonary artery to the descending aorta). When compared to the Control D7 group, MV-E/A and TV-E/A ratios were significantly lower in Clinical Treatment D7 group (*p*-values 0.04 and 0.01, respectively). No significant changes were observed in the Beta-P Infusion D7 group. PLI values, Ao-Vmax, and TCD of the treatment groups were similar to that of the control subjects (Fig. 3), which did not reach statistical significance.

#### Quantitative Polymerase Chain Reaction Analysis of Gene Expression Changes in the Fetal Heart

**Fig. 4:**
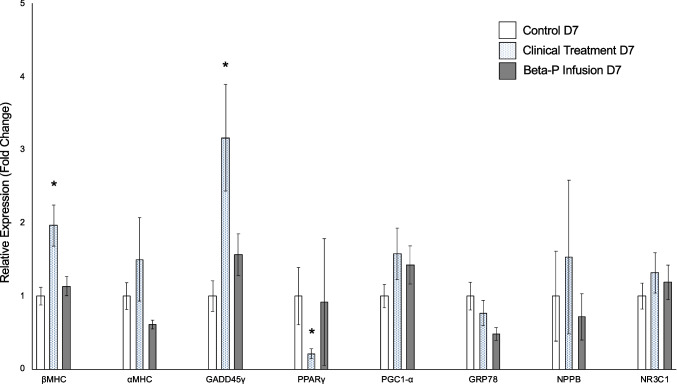
Differential mRNA expression (cardiomyocyte). The mRNA level for NR3C1 (GR) and the cardiac hypertrophic marker genes; βMHC, αMHC, GADD45γ, PPARγ, PGC1-α, GRP78, and NPPB were determined by real-time PCR and normalized to ribosome 18 s. GR indicates glucocorticoid receptor; Beta-P, betamethasone phosphate. *p < 0.05 vs. control

We sought to determine whether there were any transcriptomic changes in genes related to myocardial function or glucocorticoids evident in the steroid treatment groups. We initially performed qPCR analysis for the following genes of interest: βMHC, αMHC, GADD45γ, GRP78, and NPPB, which are shown to be related to myocardial function, and PPARγ, PGC1-α, and NR3C1, which are impacted by glucocorticoids. Significant increases were detected in the relative expression levels of β-MHC and GADD45γ, and significant decreases in PPARγ in the Clinical Treatment D7 group compared to the Control D7 group (*p* = 0.003, *p* = 0.01, and *p* = 0.03, respectively). There was no significant difference in either β-MHC, GADD45γ, or PPARγ between Beta-P Infusion D7 group and Control D7 groups. There was no significant difference in other genes (PGC1-α, NR3C1, GRP78, and NPPB) between Control D7 group and each of the steroid treatment groups (Fig. 4). Scatter plot for each of eight targets assessed by qPCR is shown in Supplementary Fig. 2.

### Microarray Analysis

**Fig. 5:**
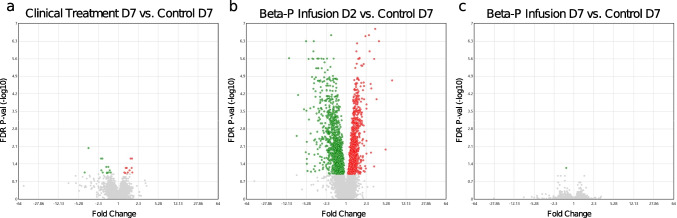
Microarray data analysis. **a**, **b**, **c** Volcano plot of each steroid treatments vs. Control D7 group (|fold change|≧1.5, *p* < 0.05; Benjamini–Hochberg multiple testing correction)

Having demonstrated the alterations in selected myocardial genes associated we obtained additional funding to further investigate global transcriptomic changes in the fetal heart tissue. Microarrays were performed on the myocardial RNA of four groups, Control D7 group, Clinical Treatment D7 group, and Beta-P Infusion D7 group, with addition of a Beta-P Infusion D2 group in order to investigate differences between acute and longer-term changes in the Beta-P infusion animals. In total, 22,141 probes were included on the microarray plate. Each treatment group was compared to the negative saline control with an FDR of 0.1. To detect smaller changes, we did not set a threshold for fold changes. Compared to the Control D7 group, the microarray analysis of gene expression identified 25 differentially expressed genes (DEGs) in the Clinical Treatment D7 group, 2375 DEGs in the new Beta-P Infusion D2 group, and, strikingly, only 1 DEG in the Beta-P Infusion D7 group, shown in the volcano plot (Fig. 5a ~ c). The raw data of microarray is shown in supplementary file.

The only DEG in the Beta-P Infusion D7 group that was included in the list of DEGs from the Beta-P Infusion D2 group was mitogen-activated protein kinase 8 interacting protein 2, which encodes a scaffold protein that is thought to be involved in the regulation of the c-Jun amino-terminal kinase signaling pathway.

To analyze the biological function of the DEGs, we further went on to perform pathway analysis using DAVID [27]. No significant pathways of interest were found in 25 DEGs in the Clinical Treatment D7 group. However, significantly regulated pathways were detected in DEGs from the Beta-P Infusion D2 group. Genes having a fold change (FC) greater than 1.5 were selected as differentially upregulated, counting up to 230 genes, and were used for the pathway analysis, revealing gene networks involved in carbon, amino acid, and fatty acid metabolism, PPAR signaling pathways, and neurological diseases. On the other hand, FC less than − 1.5 were selected as downregulated genes, containing 564 genes, and were significantly related to pathways involved in nuclear such as DNA, histone and nucleosome and epithelial cell growth factors. The top 30 gene networks obtained are shown in Table S1.

## Discussion

### Principal Findings

The purpose of the present study was to test the hypothesis that treatment with a low-concentration betamethasone phosphate infusion would have a smaller effect on fetal heart function in the acute phase and gene expression changes related to myocardial hypertrophy in the subacute phase compared to the conventional, higher-dose ACS treatment. Our data suggest that, when assessed by ultrasound 8 h after treatment commencing, the Beta-P Infusion D7 group had no significant differences in fetal cardiac function compared to Control D7 group. Additionally, we reported that more than 99% of genes differentially expressed in Beta-P Infusion D2 group were transiently altered, with just 1 significantly differentiated gene identified in the Beta-P Infusion D7 group. The conventional Clinical Treatment D7 group showed abnormal cardiac compliance by ultrasound and toxicity of some genes for markers of myocardial hypertrophy, and a greater degree of global gene expression changes.

### Cardiovascular Effects of Antenatal Corticosteroid

The short-term beneficial effects of ACS exposure on fetal cardiovascular response include increased fetal femoral vascular resistance and fetal blood pressure [28], associated with a significant reduction in the need for vasopressors [3], stabilized glomerular filtration rate [29], and improved water sodium homeostasis after delivery. Together, these changes result in improved preterm cardiovascular stability [30]. Despite the advantages listed above, ACS administration has been reported to transiently decrease the pulsatility indices in the middle cerebral artery and umbilical artery [31, 32]. No study of antenatal corticosteroids has yet been published on the ventricular diastolic function indicated by the E/A ratio.

### Clinical Implications of Cardiac Function

Although there are several reports of long-term exposure or chronic illness reducing the E/A ratio in the fetuses overexposed maternal glucocorticoid [33] and in the fetuses whose mothers had pregestational diabetes or gestational diabetes [34], there are no reports that ACS exposure rapidly reduces E/A ratio. We analyzed fetal cardiac function by ultrasound 8 h after treatment initiation when fetal plasma betamethasone concentrations were predicted to be at their maximum. It is possible that the reduction of the E/A ratio in the Clinical Treatment D7 group in the present study indicated impaired relaxation of the ventricles.

In discussing the importance of the E/A ratio in the fetus, it is first necessary to mention the development of cardiac function in the fetal period. The Doppler waveforms of the atrioventricular valves are quantified by the E/A ratio. Thus, the E/A ratio represents fetal diastolic function. The E wave component of the spectral Doppler represents passive ventricular filling in the process of myocardial relaxation. The A wave component of spectral Doppler represents active ventricular filling associated with the atrial contraction. During fetal life, diastolic filling relies mainly on atrial contraction due to the lower compliance of the fetal myocardium when compared to the adult. With advancing gestation, there is an increase of both transtricuspid and transmitral peak E but little change in peak A, resulting in a substantial increase in E/A ratio from approximately 0.5 at 13 weeks of gestation to 0.8–0.9 at 36–38 weeks’ gestation [35, 36]. This is thought to reflect the combined effects of decreasing afterload due to the development of systemic vasculature and improved diastolic ventricular compliance due to decreased myocardial stiffness and increased myocardial relaxation, which are likely to result in decrease of ventricular diastole pressures, favoring passive filling during early diastole [35].

Although it is possible that the alterations of fetal cardiovascular functions and gene expression are due to a direct effect of betamethasone [24], the underlying mechanisms responsible for the alterations in fetal circulation after ACS administration are not clear. The mechanisms producing an increase in fetal arterial blood pressure during betamethasone administration, effected via glucocorticoid receptor (GR), may be mediated by an increase in fetal cardiac output and/or an increase in fetal total peripheral vascular resistance [28]. Glucocorticoids are known to play an important role in regulating the coupling of α- and β-adrenergic receptors to post-receptor signal transduction mechanisms [28]. Glucocorticoid-induced increases in peripheral vascular resistance have been proposed due to several GR-mediated effects, such as increase in Ca^2+^ entry into vascular smooth muscle, increase in vascular sensitivity to vasoconstrictor hormones, and inhibition of prostacyclin and nitric oxide synthesis [28]. Another possible explanation might be changes in mRNA levels that compromise myocardial function in the acute phase due to a direct and dynamic role for glucocorticoids and glucocorticoid receptor signaling [24, 25, 37]. In the fetoplacental circulation, betamethasone treatment is associated with decreased placental vascular resistance, possibly induced via increased placental CRH secretion [38]. In the future, it would be desirable to observe the relationship between the diverse vasoactive effects of synthetic steroids and fetal cardiac function.

Although reduced E/A ratio may be one of the important parameters that indicate a cardiac impaired relaxation, the information offered by E/A ratios alone might be limited. Because there was no significant effect on preload findings such as TCD or PLI values, or afterload findings such as V-max in the Clinical Treatment D7 group, it was thought that there was modest impaired relaxation in the Clinical Treatment D7 group. Although the present study did not demonstrate statistical significance of afterload elevation, possibly involving increased peripheral vascular resistance [28], it may prove useful to analyze afterload elevation if the sample size was increased. When combined with other fetal function parameters and measured over time after the peak of drug administration, these data may contribute to understanding of the diastolic cardiac period in complicated fetuses [39]. Hence, intensive surveillance of fetuses with Doppler ultrasonography is warranted following betamethasone therapy. A limited number of studies have examined acute alterations of the E/A ratio due to short-term ACS exposure [40]. Therefore, we believe that additional studies are needed to determine the sequential effects of ACS exposure on cardiac function.

### Transcriptional Implications

We examined how ACS causes genetic stress response at day 7 in the subacute phase, i.e., how two treatments with different blood levels affect differently, and how myocardial stress changes in a time-dependent manner. Significant increases in the relative expression of genes for β-MHC and GADD45γ, and the significant decrease of PPARγ by qPCR in the Clinical Treatment D7 group are considered to be representative of hypertrophic alterations in many other studies as follows.

Both in vivo and in vitro studies have implicated glucocorticoids in the development of cardiac hypertrophy, a major cause of congestive heart failure [20, 24, 41]. A longstanding debate in the literature is whether or not cardiac hypertrophy can be separated into physiological and pathological types of cardiac hypertrophy [42, 43]. Pathological hypertrophy is, for example, mediated by increase in pressure overload or hypertension with the heart contracting against an increased after load. Some of its hallmarks are decreases in αMHC levels and increases in βMHC levels. In contrast, physiological hypertrophy can be induced by exercise or by increased thyroid hormone action. It is characterized by increased αMHC levels and decreased βMHC levels [42]. In detailed analyses, it appears that the effect of dexamethasone on fetal heart differs from that in the newborn heart in terms of αMHC expression, namely, decreased expression following fetal exposure but increased expression by the neonatal heart ^41^ [44]. In addition, an increase in βMHC is associated with cardiac hypertrophy in fetuses [24, 42, 43]. Reduced fetal growth due to maternal nutrient restriction [44], maternal dexamethasone treatment [45], and the induction of ventricular hypertrophy in rats due to hypertension [46] can each result in altered expression of cardiac genes involved in energy metabolism. While peroxisome proliferator-activated receptors (PPARs) are involved in energy metabolism and mitochondrial function-related genes, multiple studies have shown the important role of PPARγ in the cardiac hypertrophy [47]. Yamamoto et al. reported that PPARγ activators inhibit cardiac hypertrophy in cardiac myocytes [48]. Wyrwoll et al. also have showed the effects of overexposed maternal glucocorticoids on reduced αMHC and PPARγ in fetal cardiomyocytes of mice [33]. The growth arrest and DNA-damage-inducible 45 family include three isoforms, α, β, and γ (Gadd45γ), known to elicit pleiotropic effects, inducing cell cycle arrest, DNA repair, and ultimately promoting apoptosis in response to physiological stress. Lucas et al. have reported that the GADD45γ overexpression in cardiomyocytes could affect the activation of p38 MAPK signaling pathway and be associated with the development of heart failure [49].

In the present study, the fetuses in the Clinical Treatment D7 group showed significantly increased βMHC expression levels which implicated pathological hypertrophy in cardiomyocytes, but did not show differential αMHC expression. In addition, decreased PPARγ and increased GADD45γ in the fetuses in the Clinical Treatment D7 group indicated hypertrophic stress on fetal cardiomyocytes. The microarray results show that fetuses in the Beta-P Infusion D2 group that were treated for 2 days showed functional changes in energy metabolism and the PPAR signaling pathway. We examined the effect of clinical ACS on GADD45γ’s hyperexpression for the first time. Therefore, future studies of the effects of ACS on gene expression and protein production in fetal myocardium may focus on myosin-actin, energy metabolism (including the PPAR family), and GADD45γ.

## Current Issues and Future Prospects

With regard to the programming effect on the fetal heart exposed to ACS, it has been reported that there was an effect on the development of the cardiac autonomic nervous system, which had a stronger effect on females and a weaker effect on non-Caucasians [23]. Mzayek et al. examined the association between ACS exposure and cardiovascular risk factors for Caucasians and African-Americans aged 7 to 21 years and noted racial differences [50]. Gabory et al. pointed out that recent studies have shown that fetal developmental programming is finely tuned for each sex by the effects of gametogenesis, sex steroid hormones, and sex chromosomes, and that epigenetic effects that are transmitted three generations down the line are transmitted in a sex-specific manner [51]. As a new perspective, we have also studied the causes of individual differences in responsiveness to steroids that exist between sheep fetuses [52]. Thus, it should be kept in mind that the effects of ACS exposure may vary among individuals, including gender and race, and that it is likely necessary to personalize ACS doses and to take into account the fact that long-term adverse effects of ACS exposure may also vary among individuals.

Although this study examined the optimization of ACS treatment by continuous infusion of Beta-P from the perspective of the fetal heart, it must be said that continuous infusions of Beta-P likely have low therapeutic value due to cost and inconvenience. Patient compliance with ACS treatment based on a slow-release patch or oral medication may be alternative means of achieving low-dose extended exposures that achieve good lung maturation. A study of low-dose ACS treatment with oral medication in pregnant sheep demonstrated fetal lung maturation [53, 54], and a study in pregnant monkeys showed that low-dose ACS treatment resulted in efficient fetal lung maturation and had less effect on the hippocampus than clinical treatment with intramuscular injection [55].

### Limitations

It is important to note that the low-dose betamethasone animals used in this study underwent a minor fetal recovery surgery procedure, whereas the higher dose steroid animals and saline control animals received intramuscular injections only. Assuming that fetal surgery conveys a greater risk of cardiac dysfunction, it is possible that the study results underestimate the difference in cardiac effects between a high-dose exposure delivered by intramuscular injection and constant low-dose fetal steroid exposure. The limitations of the present study include a relatively small sub-group size. Due to the need to perform ultrasound assessments at tightly controlled intervals (i.e., 8 h after treatment initiation), we were somewhat limited in the number of animals that we could image. We decided that a tighter grouping of animals at the 8-h post-treatment interval was more advantageous than a larger number of animals imaged over a wider interval—potentially introducing additional noise to the study. Future experiments will be stratified so as to allow the capture of ultrasound data from a larger cohort of animals.

We assumed two things in the present study: that pretreatment cardiac function would be similar in all groups, and that the greatest change in cardiac function would occur after 8 h on the day of treatment, when betamethasone blood concentrations were at their maximum. Therefore, we performed ultrasound examinations only on the day of treatment. With these data in hand, additional time-course studies are warranted and can now be designed to explore temporal and individual subject effects.

In the present study, we were unable to preserve myocardial tissue from the Clinical Treatment D2 group. With these tissues, comparisons of microarray results between the Beta-P Infusion D2 and Clinical Treatment D2 groups would have been possible.

Although this study investigated myocardial dysfunction using ultrasound and transcriptomic techniques, a host of additional analyses can be considered for future experiments.In particular, pathological evaluation and proteomic analysis of the myocardium may be useful in assessing myocardial damage, as Severinova et al. reported in rats [25].

## Conclusions

In the present study, treatment with a clinical ACS regimen elicited greater acute perturbations in fetal cardiac function than treatment with a low-dose regimen. At a transcriptional level, the use of low-dose betamethasone elicited transient changes in cardiac mRNA expression at 2 days post-treatment, and these changes were almost entirely resolved by 7 days. In contrast, more genes remained differentially regulated at 7 days post-treatment in the clinical ACS treatment group. Although the clinical treatment of antenatal corticosteroid significantly affected cardiac diastolic function in acute phase and some hypertrophic actions in myocardium in fetus in subacute phase, the low-dose Beta-P exposure had less influences on those.

These results suggest that a low-dose steroid exposure may reduce the risk of adverse impacts on the developing fetal heart. Future studies are needed to determine the long-term impact of steroid exposures on the fetal heart, and the potential for lower dose ACS treatments to reduce the risk of harm.

### Supplementary Information

Below is the link to the electronic supplementary material.

Supplementary file1 (XLSX 11.7 KB)

Supplementary file2 (XLSX 3.56 MB)

Supplementary Fig. 1 ESM 1 (PNG 191 kb)

High resolution image (EPS 74.1 kb)

Supplementary Fig. 2 ESM 1 (PNG 75.2 kb)

High resolution image (EPS 88.4 kb)

## Data Availability

All data supporting the findings of this study are available within the paper including supplementary file.
