# Supplementary figures and images for: A Reduction in Antenatal Steroid Dose Was Associated with Reduced Cardiac Dysfunction in a Sheep Model of Pregnancy
Source: Reprod Sci. 2023 Jun 1;30(11):3222–34. doi: 10.1007/s43032-023-01264-2 (PMC10643432; doi:10.1007/s43032-023-01264-2)

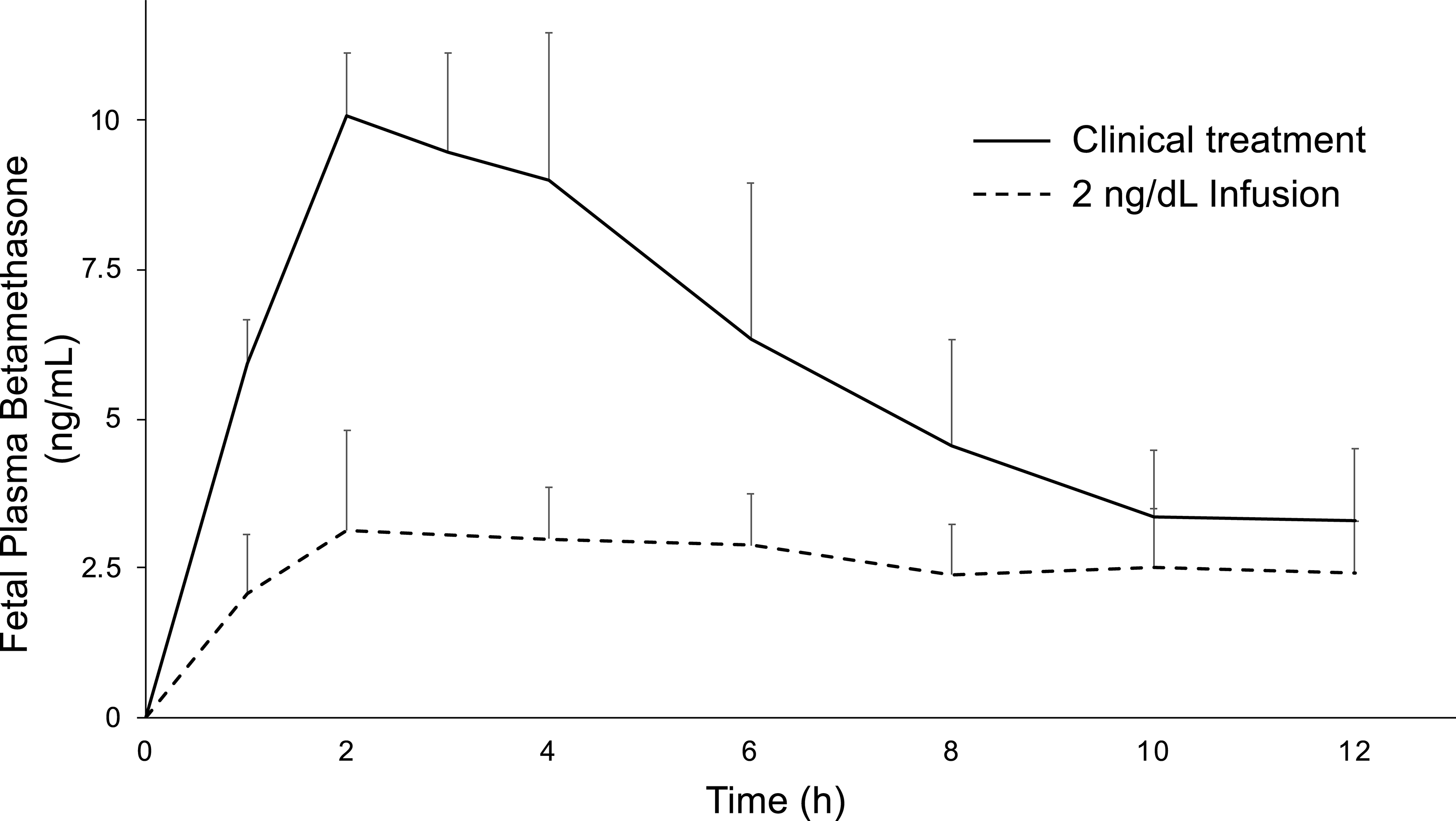

Supplement: Supplementary file 3 — ESM 1 (PNG 191 kb) [file 43032_2023_1264_Fig6_ESM.png]

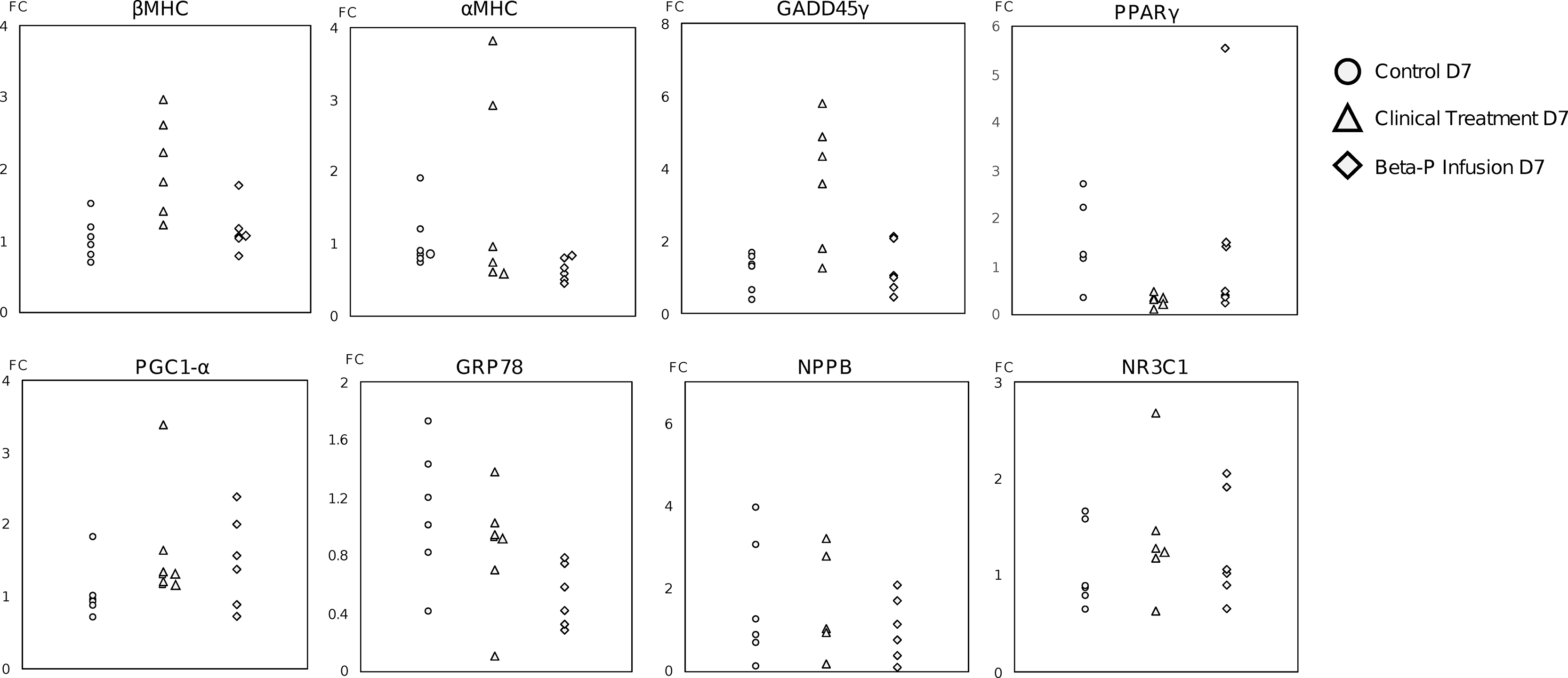

Supplement: Supplementary file 5 — ESM 1 (PNG 75.2 kb) [file 43032_2023_1264_Fig7_ESM.png]
